# Nutritional Deficiencies in Radiotherapy-Treated Head and Neck Cancer Patients

**DOI:** 10.3390/jcm10040574

**Published:** 2021-02-03

**Authors:** Tomasz Powrózek, Joanna Dziwota, Teresa Małecka-Massalska

**Affiliations:** Department of Human Physiology, Medical University in Lublin, Radziwiłłowska 11, 20-080 Lublin, Poland; dziwota.joanna@gmail.com (J.D.); tmalecka@gmail.com (T.M.-M.)

**Keywords:** head and neck cancer, cachexia, sarcopenia, malnutrition, body composition

## Abstract

Nutritional deficiencies (malnutrition, cachexia, sarcopenia, and unfavorable changes in the body composition) developing as a side effect of radiotherapy (RT) currently represents a significant but still inaccurately studied clinical problem in cancer patients. The incidence of malnutrition observed in head and neck cancer (HNC) patients in oncological radiology departments can reach 80%. The presence of malnutrition, sarcopenia, and cachexia is associated with an unfavorable prognosis of the disease, higher mortality, and deterioration of the quality of life. Therefore, it is necessary to identify patients with a high risk of both metabolic syndromes. However, the number of studies investigating potential predictive markers for the mentioned purposes is still significantly limited. This literature review summarizes the incidence of nutritional deficiencies in HNC patients prior to therapy and after the commencement of RT, and presents recent perspectives for the prediction of unfavorable nutritional changes developing as a result of applied RT.

## 1. Introduction

### 1.1. Head and Neck Cancer

Head and neck cancer (HNC) consists of a group of malignant neoplasms, mainly squamous-cell carcinomas (approx. 90% of tumors), that are heterogeneous with regard to their anatomical location, etiology, and clinical presentation, occurring in the mouth, throat, larynx, salivary glands, paranasal sinuses, and ear [1,2]. HNC forecasts are unfavorable, predicting a systematic increase in the number of cases and deaths. In the United States, over 65,000 new HNC cases and 14,500 deaths were estimated for 2020, which constitutes a 33.3% and 28.8% increase, respectively, compared to 2010 [3,4,5,6].

HNC is characterized by an unfavorable prognosis and the percentage of 5-year survival in this group of patients, regardless of the stage of cancer progression, which also depends on the anatomical location of the tumor, its degree of differentiation, the treatment used, and the clinical-demographic characteristics of the patient, does not usually exceed 30–40% [7,8]. In addition, development of the disease is promoted by risk factors to which residents of both developing and developed countries are exposed. The most important ones are smoking tobacco, regular alcohol consumption, and oncogenic human papilloma virus (HPV) infection resulting from risky sexual behavior and genetic alterations [9,10,11,12,13].

The choice of the HNC treatment method depends on the anatomical location, clinical stage, and histological differentiation of the tumor, as well as the clinical and demographic features of the patient, including age, fitness, the presence of comorbidities, and the nutritional status. In addition to surgical treatment, radiotherapy (RT) constitutes essential and routine HNC treatment, and in advanced stages of the disease, it is often supplemented with chemotherapy (CRT). If radical RT is used as the sole method of treatment or in combination with chemotherapy, conventional radiation doses are used (1 fractional dose: 1.8–2.0 Grey (Gy)/day for 5 days a week in a treatment regimen lasting 5–7 weeks (total dose of about 70 Gy)). This allows the achievement of therapeutic effects comparable to surgical treatment and permits the organ affected by cancer to be saved. Currently, in order to reduce RT complications, save healthy tissue, and precisely adjust the radiation dose delivered to the tumor tissue, Intensity Modulated Radiation Therapy (IMRT) is used [14,15,16,17].

Regardless of the applied RT scheme and the obtained treatment results, RT is associated with a high risk of developing early or late radiation reactions. These adverse effects can be more fatal in the case of CRT. Moreover, various degrees of toxicity are observed in all patients who have completed the full course of treatment. Serious physical symptoms and functional disorders and losses caused by the disease and its treatment using RT have negative clinical, psychological, and social effects [18,19]. The infiltration of anatomical structures located in the common pathway of the digestive and respiratory systems, impairment of the functions of the sensory organs, and damage to the structures responsible for food intake caused by ionizing radiation lead to the intensification of catabolic processes in the body and a decrease in the energy supply. As a consequence, the above-mentioned disorders may lead to the development of malnutrition and/or cachexia, which are serious clinical problems related to deterioration of the patient’s general condition, a worse response to treatment, and a shorter survival time [18,20].

### 1.2. Malnutrition, Cachexia, Sarcopenia and Changes in the Body Composition in the Course of Cancer—Definition and Pathophysiology

Currently, the definition of malnutrition, cachexia, and sarcopenia is the subject of lively discussions among researchers of various specialties. A multifactorial background leading to the development of the two metabolic disorders, including abnormalities in the functioning of biochemical, molecular, and immunological pathways, effectively prevents the achievement of the desired consensus [21,22]. According to the clinical guidelines of The European Society for Clinical Nutrition and Metabolism (ESPEN) and Society on Sarcopenia, Cachexia and Wasting Disorders (SCWD), elaborated on the basis of experience and clinical observations, malnutrition developing in the course of neoplastic disease can be defined as a clinical condition that results from systemic inflammation caused by the underlying disease (cancer) and characterized by an imbalance of energy, protein, and other nutrients that results in measurable changes in the body composition, weight loss, and deterioration of the body’s physical activity [23,24,25]. Cachexia (wasting syndrome) is a multifactorial syndrome characterized by unintentional weight loss with a progressive loss of muscle mass with or without adipose tissue loss that cannot be completely reversed by conventional nutritional therapy. In addition, cachexia is characterized by a negative protein and energy balance caused by a disturbed food intake and abnormal metabolism featuring increased energy expenditure, insulin resistance, lipolysis, and proteolysis, which intensify weight loss and are induced by systemic inflammatory factors. Untreated malnutrition may progress to cachexia, and the first symptom of this is often the loss of muscle mass. It is worth noting, however, that not all patients suffering from malnutrition develop cachexia, while all patients with cachexia develop malnutrition to varying degrees [26,27,28]. Sarcopenia is a condition characterized by the loss of muscle mass and muscle strength. The development of sarcopenia may be associated with conditions that are not exclusively seen in older persons, such as malnutrition and cachexia, though sarcopenia is primarily a disease of the elderly. The loss of muscle mass is typical of cachexia, but most sarcopenic individuals are not cachectic. Patients with no weight loss, a lack of anorexia, or a systemic inflammatory response may well be sarcopenic [28,29].

**Figure 1:**
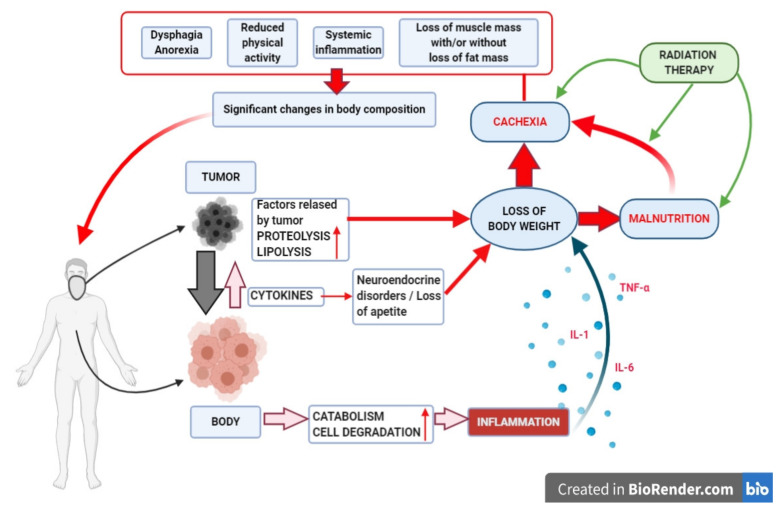
The mechanism of malnutrition and cachexia in neoplastic diseases postulated by most researchers. The classic definition was complemented by the unfavorable impact of radiotherapy (RT) on the nutritional status of the head and neck cancer (HNC) patients (created with the BioRender tool [38]).

Following the initiation of the neoplastic process and its further progression, the key function of the body, which is nutrition, becomes impaired. In the physiological state, nutrition is precisely controlled by mechanisms regulating hunger, satiety, and molecular and biochemical pathways responsible for the supply of substances that provide energy to tissues and then their use for the needs of intracellular metabolism. The proper functioning of the above mechanisms allows the body to maintain a dynamic balance between the processes of energy-providing substance consumption and their use for cellular metabolism, as well as the storage of their surplus (catabolism-anabolism balance) [30,31]. The disturbance of said balance by changing cellular metabolism in favor of catabolic processes leads to a gradual disruption of the quantitative and qualitative composition of energy-providing substances in the body, which leads to an impaired function of cells, tissues, and organs, and, consequently, the whole body, with effects of varying intensity occurring in cancer patients [32]. In light of recent studies, disorders of the catabolic-anabolic balance in the course of neoplastic disease are attributed to both the developing cancer and the body of the patient trying to defend themselves against the “intruder”. The developing tumor initially mainly consumes the energy substrates circulating in the host’s blood—carbohydrates, fats, and proteins—to satisfy hyper anabolic processes that enable rapid and uncontrolled cell proliferation and, hence, a tumor mass increase. Along with the disease progression in the patient’s body, the cancer’s metabolic needs also increase, which requires the supply of increasing amounts of energy and building materials [33]. At this stage, the energy needs of the tumor can only be meet by the release of fats and proteins stored in the patient’s adipose and muscle tissues under the influence of lipolytic and proteolytic factors secreted by cancer cells [34]. On the other hand, the body, which is being gradually cut off from the supply of energy-providing substances, is forced to cover its own energy requirements at the expense of a further loss of adipose and muscle tissues. In addition, in response to the developing pathology, the patient’s body produces a number of pro-inflammatory cytokines (IL-1, IL-6, and TNF-α) [35]. Although they act as alarm and defense mechanisms of the body, their long-term release and persistently high level in the body lead to a negative effect on adipose and muscle tissue, as well as liver and brain functions. Currently, many researchers postulate the development of generalized inflammation as one of the key mechanisms leading to the development of malnutrition and cachexia [36,37]. According to clinical observations and the confirmed adverse effect of the applied therapy, the current definition of nutritional disabilities should be supplemented by the unfavorable effect of the therapy (surgery, chemotherapy, RT, and CRT) on the nutritional status of cancer patients. The mechanism of malnutrition and cachexia in neoplastic diseases postulated by most researchers and definition complemented by the impact of RT on the nutritional status of the HNC patients are presented in Figure 1.

The result of the above-described metabolic disorders developing under the influence of the ongoing cancer process is disruption of the body’s caloric balance, the development of inflammation, a loss of cell mass, and a change in the body composition that can lead to the development of malnutrition or wasting of the body [39,40].

## 2. Nutritional Deficiencies in HNC Patients

### 2.1. The Problem of Malnutrition in HNC Patients

For decades, the presence of cancer cachexia was considered an obvious consequence of the ongoing neoplastic process in the body. Over the years, however, significant differences have been observed in the response to treatment and the survival of patients suffering from cancer cachexia compared to patients with a normal nutritional status, despite similar clinical-demographic characteristics. Unlike cachexia, sarcopenia was previously matched with an older age. Nevertheless, 22.5% of cancer patients present a risk for sarcopenia. Alarmingly, the prevalence of sarcopenia in HNC patients has been demonstrated to be high, although there is considerable between-study variation (16–71%). We now know that malnutrition, sarcopenia, and cachexia in cancer patients constitute an unfavorable clinical factor associated with deterioration in the quality of life and a worse response to the applied therapy, as well as a shorter survival time [41]. Malnutrition of various degrees is reported in 50–80% of cancer patients, and about 20–30% of patients in the terminal stage of the disease do not die of cancer, but due to long-term wasting of the body, which is no longer able to support the functions of vital organs due to the depletion of energy substrates [42,43].

HNC patients are at a very high risk of developing malnutrition, and about 60,000–90,000 patients die from cancer cachexia every year. The high rate of malnutrition in this group of patients is affected by the anatomical location of the tumor, the degree of its infiltration of the structures responsible for providing food to the body, and the toxicity of the applied therapy [42,44]. Approximately 50–70% of HNC patients are diagnosed with malnutrition of varying degrees, and progressive weight loss is often one of the first visible signs of cancer [45]. Although there are diagnostic tools based on clinical scales (Subjective Global Assessment—SGA and Malnutrition Universal Screening Tool—MUST), anthropometric measurements (BMI), electrical bioimpedance (BIA), or dual energy X-ray absorptiometry (DXA), which are able to detect malnutrition, sarcopenia, or cachexia with various degrees of sensitivity and specificity before or after treatment, there are still no objective predictive markers that would allow patients at the highest risk of developing malnutrition or cachexia during therapy to be initially selected [46,47,48]. The prevalence of malnutrition in RT-naïve HNC patients is summarized in Table 1.

### 2.2. Nutritional Deficiencies in HNC Patients Treated with RT

Malnutrition, cachexia, and sarcopenia developing as a side effect of cancer treatment is currently a significant but still inaccurately studied clinical problem in cancer patients. RT or CRT, which are characterized by a high aggressiveness in the destruction of tumor tissue, unfortunately also damage healthy tissues, which results in either the development of malnutrition or intensification of the already existing malnutrition, leading to cachexia [61,62]. The negative effect of therapy on the nutritional status of HNC patients is confirmed by the high percentage of malnutrition (44–88%) found after the completion of treatment in this group of patients. RT toxicity leads to gastrointestinal disorders, such as vomiting, diarrhea, xerostomy, stomatitis, and taste disorders, as well as a loss of appetite and anorexia. In addition, the side effects of RT or CRT are associated with a negative impact on the patient’s mental condition. Patients may feel anxious or unwilling to eat as they associate eating with physical pain that accompanies biting, chewing, and swallowing [21,63]. These side effects of RT lead to a significant reduction in the supply of food and energy. This promotes the intensification of catabolic processes within the organism, which results in a gradual loss of body mass and its remodeling (changes in the body composition) associated with progressive proteolysis and/or lipolysis of muscle and/or fat tissue [64,65]. The most recent meta-analysis conducted demonstrated an unfavorable impact of RT-based therapy on sarcopenia incidence; its prevalence ranged from 6.6 to 64.6% pre-treatment and 12.4 to 65.8% post-treatment [66]. However, the incidence of malnutrition observed in HNC patients in oncological radiology departments can reach 80% [67,68]. The presence of malnutrition, cachexia, and sarcopenia is associated with an unfavorable prognosis of the disease, a higher mortality, and deterioration of the quality of life. Therefore, it is necessary to identify patients with a high risk of the mentioned metabolic syndromes [68]. The problem of malnutrition developing as a result of treatment and its impact on the patient’s life and treatment results is so important that, based on the above clinical observations, the classical definition of malnutrition and cachexia has been expanded to also include other factors (the applied therapy) conducive to their development beyond the factors related to the presence of a tumor and metabolic disorders in the body. It is believed that any involuntary weight loss ≥ 5% within 1 month is a reliable indicator of malnutrition associated with hospitalization and the applied treatment [69,70]. This emphasizes that the therapy and adverse effects associated with it may significantly increase the dynamics of malnutrition development, even in short periods of time, such as the duration of radical RT (5–7 weeks). The prevalence of nutritional deficiencies developing in the course of RT ispresented in Table 2.

### 2.3. Prediction of Nutritional Deficiencies Developing during the RT Course

Assessment of the risk of cancer malnutrition, sarcopenia or cachexia at the stage of RT planning in HNC patients seems to be crucial for determining the patient’s further prognosis, the success of the applied therapy, and the risk of early and long-term effects of its toxicity [88]. The currently available predictive tools—the patient’s clinical features (age, smoking, and socioeconomic status), anthropometric measures (body weight, and BMI), clinical scales (Nutritional Risk Score—NRS-2002), or laboratory tests (markers of inflammation and the albumin level)—are insufficient for predicting the development of malnutrition during RT [54,67,81,89,90,91]. The more reliable tools for the prediction of RT-induced changes in body composition demonstrate parameters derived from BIA, mainly the phase angle (PA), whose value is decreased in malnourished/cachectic patients [92]. For cachexia and sarcopenia detection, the most reliable tools are computed tomography (CT) and DXA [90,91]. The skeletal muscle index (SMI) is a measure of sarcopenia that can be obtained from diagnostic imaging studies, mainly CT. SMI measurement can be used globally to select patients for potential suitable therapy, and patients with a low SMI are more likely to be sarcopenic. Patients with a low SMI also had a significantly poorer prognosis than others, especially those who received definitive RT. SMI measured prior to RT can serve as a prospective biomarker of RT-induced sarcopenia. By establishing the optimal cut-off value, the changes in SMI noted during the therapy course can be considered as alternatives for the diagnosis of sarcopenia in routine examinations (ROC value > 0.9) [93,94,95]. In light of recent research in the field of genetics and molecular biology, more attention is being paid to molecular markers (gene polymorphisms, the expression of non-coding RNA, and epigenetic alterations), which can be very useful for diagnostic and predictive and prognostic purposes in cancer patients [96]. However, in the literature to date, there are very limited study results assessing the predictive value of molecular markers of malnutrition and cachexia developing during radical RT treatment in cancer patients, including HNC patients. The high application potential of the above markers also highlights the need to test their ability to detect and predict malnutrition, cachexia, and changes in body composition, while assessing the disturbance of their function (intensification or weakening) seems to be key to understanding the pathological mechanism of the development of both metabolic syndromes. This is justified by the molecular background leading to malnutrition, including the development of inflammation and fat and protein metabolism disorder as a result of increased lipolysis and proteolysis leading to quantitative and qualitative changes in the body composition [97]. The above-described processes are controlled by proteins, which demonstrate differences in the level of their activity as a result of the presence of, among others, polymorphisms in the genes encoding them or disorders in the mechanisms controlling their expression (microRNA and long non-coding RNA (lncRNA) expression). The intensification of the above mechanisms may be the result of the presence of a tumor, the patient’s response to the pathological condition, or induced by the applied therapy. To date, many single nucleotide polymorphisms (SNPs) that regulate the activity of proteins involved in the development of inflammation and the regulation of metabolic pathways of sugars, fats, and proteins have been identified, and their presence may predispose patients to the development of cancer malnutrition or cachexia [98]. Recent studies have also proven the key role of microRNA molecules in regulating fat metabolism and, above all, in the mechanism of muscle atrophy and regulation of the severity of the inflammatory response in the body [99,100]. Moreover, RT affects the changes in the microRNA expression signature measured prior to and after the commencement of therapy [101]. Non-anthropometric factors demonstrating prospective utility in the prediction of nutritional deficiencies developing during RT in HNC patients are summarized in Table 3.

Elevations in inflammatory cytokines and impairments in leptin/ghrelin functioning are associated with symptoms of cancer cachexia. Moreover, leptin level can decrease after the commencement of RT [108]. In another study, leptin increased cell proliferation and migration, as well as the colony-forming ability, despite the suppressive effect induced by RT [109]. These findings suggest that “hunger hormones” can also be attractive and prospective markers of post-RT nutritional deficiencies. Based on recent clinical experience, it is important to assess a patient’s susceptibility to developing nutritional deficiencies in the course of RT. Therefore, detailed nutritional screening is required during therapy planning. Apart from molecular markers and body composition analysis, clinical factors are still useful for cachexia prediction. Recent studies demonstrated that patients with an older age (>70 years), loss of appetite, swallowing difficulty, poor performance status, and high Nutritional Risk Score (NRS) are at a higher risk of cachexia. Regarding sarcopenia in RT-treated HNC patients, it was found that sarcopenic individuals were more likely to be older (66 vs. 62 years, *p* < 0.001) or have a worse performance status (according to ECOG-WHO), and they were less likely to have a tumor located in the larynx, stage I or II disease, or p16-positive oropharyngeal cancer [110]. Clinical symptoms along with body composition evaluation and molecular marker assessment could allow a detailed insight into a patient’s condition and should be considered during nutritional screening prior to therapy. It could exclude selected patients from therapy, for whom the therapy complications could be worse than the benefits [111,112]. The assessment of molecular changes seems to be primarily useful because of its ability to objectively reflect the body’s condition at the cellular level, including the nutritional status and mechanisms controlling this process. Cellular metabolism disorder and damage to healthy tissues exacerbated by the effect of ionizing radiation can be noted much earlier at the molecular level, days or even weeks ahead of the appearance of clinical symptoms of malnutrition.

### 2.4. Nutritional Support in RT-Treated HNC Patients

There are limited data concerning the predictive value of nutritional support in RT-treated HNC patients, because most studies have focused on the treatment methods of post-RT nutritional deficits. However, according to nutritional management guidelines, nutritional intervention should be part of the management of RT-treated HNC patients. Nutritional intervention should be tailored to meet the needs of the patient and be realistic for the patient to achieve. Individualized nutritional intervention either during planning or for early stage RT may be beneficial in terms of decreasing the impact of its side effects, as follows: Decreasing unintended weight loss; improving dietary intake and quality of life; reducing acute toxicities and treatment interruptions; and positively affecting the survival. It is recommended that nutritional intervention takes place before RT is started and continued during and after treatment [113,114]. For these goals, the three main methods of nutritional support are oral, enteral, and parenteral. Parenteral nutritional support is rarely used in the HNC setting; however, it should be considered if required [115]. Current guidelines from the ESPEN recommend that ambulating patients with HNC should receive 1.2 to 2 g/kg/day of protein and 30 to 35 kcal/kg/day of energy daily [116]. Truly, such timely nutritional intervention can improve the curative effect for patients undergoing RT by the effective prevention of weight loss and muscle wasting. In a recent study concerning the energy intake in RT-treated HNC patients, it was found that an increased energy intake during RT can reliably reduce the post-treatment prevalence of severe malnutrition and increase the number of well-nourished individuals [55]. Prospective and retrospective trials in HNC undergoing RT demonstrated that enteral nutrition compared with oral feeding reduces weight loss, the frequency and duration of treatment interruptions, and the rate of hospital admission [117,118]. In HNC patients treated with either RT or CRT, individualized dietary counseling was beneficial for the quality of life, but the impact of tube feedings was not conclusive [119]. The latest findings also suggest a significant role of controlled physical exercises and rehabilitation in the curation of post-RT malnutrition, sarcopenia, and cachexia. However, a meta-analysis did not confirm the beneficial combination of nutritional support with controlled physical activity regarding patients’ nutritional benefits [112]. Moreover, dietetic counseling alone or associated with supplementation by enteral nutrition for three months was not able to prevent a loss of muscle strength and body weight during RT [113].

## 3. Conclusions

Malnutrition, cachexia, sarcopenia, and body composition changes are some of the major issues in RT-treated HNC patients. Unfortunately, there are a lack of established clinical guidelines allowing the successful management of HNC patients undergoing RT intervention. Data from oncology departments of European hospitals prove that only 30–60% of cancer patients at risk of malnutrition receive nutritional support in the form of oral supplementation, as well as parenteral or enteral nutrition [68]. By selecting a risk group using a molecular analysis of patients who have a high chance of development or progression of malnutrition during RT, it may be possible to make decisions regarding the introduction of nutritional treatment at the planning stage of therapy or parallel to treatment. In addition, using molecular markers to select patients at a high risk of developing both malnutrition syndromes during RT may help answer the questions of why some patients do not receive a full course of treatment (disqualification during treatment), the results of treatment are unsatisfactory, and the percentage and severity of complications are higher than expected. The development of treatment-induced malnutrition or cachexia may also be an unfavorable factor associated with the risk of early death, despite radical treatment. Molecular markers can be evaluated in a non-invasive manner based on blood sample testing, which may allow the assessment or monitoring of the patient’s nutritional status at various stages of the therapeutic procedure. Determining the specific genotype and changes in the level of expression of microRNA and lncRNA circulating in blood in patients with HNC at the therapy planning stage can be a reliable and clinically attractive non-invasive predictive marker of malnutrition and/or cachexia, which may develop during RT before the first clinical symptoms occur. Early nutritional intervention can improve the curative effect for patients undergoing RT by the effective prevention of weight loss and muscle wasting.

## Figures and Tables

**Table 1:** Prevalence of malnutrition, sarcopenia and cachexia and clinical tools used for the detection of nutritional deficiencies in therapy naïve HNC patients.

Study	Studied Group	Prevalence of Nutritional Deficiencies	Nutritional Screening	Other Major Study Findings
Jager- Wittenaar et al. [49]	26 newly diagnosed patients (69% in stage T3-T4) Major tumor site: Oropharynx (39%) and oral cavity (35%)	pre-cachexia—15% cachexia—42% nutritional deficits in 57% of patients	Anthropometric measurements: Weight loss > 5% in the past 6 months or BMI < 20 kg/m^2^ and any degree of weight loss > 2% Radiologic: DXA-Appendicular skeletal muscle index, plus any degree of weight loss > 2%	No differences in inflammatory markers were observed between cachectic and non-cachectic patients
Richey et al. [50]	24 newly diagnosed patients (70.8% in stage IV) Major tumor site: Oropharynx (50%) and larynx (25%)	cachexia—46%	Clinical inventory: M.D. Anderson Dysphagia Inventory (MDADI) and the Functional Assessment of Anorexia/Cachexia Therapy (FAACT) Anthropometric measurements: Experienced weight loss exceeding 5% of the pre-morbid body weight over 3–6 months	Clinical characteristics associated with cancer cachexia in HNSCC were T4 status, increased CRP, decreased hemoglobin, and increased IL-6. Quality of life was substantially reduced in patients with cachexia compared to non-cachectic patients
Orell- Kotikangas H et al. [51]	65 newly diagnosed patients (81.5% in stage III-IV) Major tumor site: Oropharynx (35.4%) and oral cavity (18.5%)	cachexia—31% nutritional deficits in 34% of patients	Clinical scale (PG-SGA) Anthropometric measurements: BIA Functional test: Handgrip strength (HGS)	One third of HNC patients are already cachectic at diagnosis. By combining low functional status and low muscle mass, regardless of weight loss and inflammatory status, there is an ability to show an association between cachexia and poor survival of patients
Małecka- Massalska et al. [52]	75 newly diagnosed patients (all patients in stage III-IV) Major tumor site: oropharynx (39%), oral cavity (24%), and larynx (37%)	moderate or severe malnutrition—40%	Clinical scale (PG-SGA) Anthropometric measurements: BIA	The ratio of extracellular mass to body cell mass (ECM/BCM) can be an indicator that detects malnutrition in patients with HNC
Stegel et al. [53]	55 newly diagnosed patients (96.4% in stage III-IV) Major tumor site:Oropharynx (45.5%) and larynx (20%)	cachexia—14.5% malnutrition—16.4% nutritional deficits in 30.9% of patients	Clinical scale (NRS-2002) Anthropometric measurements: Laboratory tests BIA	Phase angle (PA) derived from BIA is promising factor reflecting nutritional status of HNC patients; however, it did not distinguish between malnourished and cachectic patients with HNC
Kang et al. [54]	20 newly diagnosed patients (77.5% in stage T3-T4)	moderate malnutrition—45% severe malnutrition—35% nutritional deficits in 80% of patients	Clinical scale (PG-SGA)	HNC patients are most malnourished, which impacted clinical outcome. Timely nutritional intervention can effectively prevent weight loss and muscle wasting
Yanni et al. [55]	152 newly diagnosed patients (60.5% in stage T3-T4) Major tumor site: Oropharynx (25%), oral cavity (29.6%), and larynx (31%)	moderate malnutrition—62.6% severe malnutrition—18.7% nutritional deficits in 81.3% of patients	Clinical scale (NRS-2002) Anthropometric measurements	Tumoral, nutritional. and treatment characteristics seem to be predictors for malnutrition. These factors should be integrated in the nutrition algorithm approach
Ling et al. [56]	113 patients (all patients in stage III-IV) Major tumor site: Oropharynx (22.1%) and oral cavity (49.6%)	moderate malnutrition—21.2% severe malnutrition—0.9% nutritional deficits in 22.1% of patients	Clinical scale (PG-SGA) Anthropometric measurements Radiologic: DXA	HNC patients with nutrition risks need to be identified as early as possible to prevent malnutrition and its consequences, such as treatment delays, interruptions, and poor treatment outcomes
van Bokhorst–de van der Schueren et al. [57]	44 patients with previously non-treated tumor (all patients in stage T2-T4) Major tumor site: Oral cavity and larynx	Malnutrition based on weight loss > 10%, previous 6 months—30%, and NI score—59%	Laboratory tests: Albumin level weight loss during the past six months (PWL) Nutritional index score (NI)	Patients with more than 10% weight loss during the 6 months prior to surgery were at great risk for the occurrence of major postoperative complications
Kay et al. [58]	50 newly diagnosed patients (84% in stage III-IV) Major tumor site: Nasopharynx (52%)	moderate malnutrition—36% severe malnutrition –20% nutritional deficits in 56% of patients	Clinical scale (PG-SGA)	A significant positive relationship between Nutrition Impact Score (NIS) and PG-SGA was found, indicating the lower the NIS, the better the nutritional status among HNC patients
Silva et al. [59]	132 newly diagnosed male patients (78.9% in stage III-IV) Major tumor site: Oropharynx (33.8%) and oral cavity (25.7%)	sarcopenia—45%	Skeletal muscle mass index (SMMI) Functional test: Timed up and go test (TUG) and HGS	Prevalence of sarcopenia was higher in patients suffering from dysphagia (55%) than in non-dysphagic patients (30%)
Ganju et al. [60]	246 newly diagnosed and post-operative (81% in stage IV) Major tumor site: Oropharynx (63%)	sarcopenia—58.1%	Skeletal muscle index (SMI) Radiologic: Computed tomography (CT)	Sarcopenia is associated with radiation treatment breaks greater than 1week

**Table 2:** Nutritional deficiencies developing after RT intervention in HNC patients recorded throughout clinical studies.

Study	Studied Group	Radiation Method	Nutritional Deficiencies as a Result of RT
Malnutrition or Cachexia			
Stegel et al. [53]	55 patients	postoperative RT (43.6%) definitive RT (56.4%) chemoradiation (CRT) (69%)	Before RT, 45% of patients were moderately malnourished and 35% severely malnourished; post-treatment proportions were 40% and 50%, respectively
Kang et al. [54]	20 patients (mostly locally advanced disease)	RT (single, CRT, or adjuvant)	Before RT, 16.4% of patients were malnourished and 14.5% cachectic; post-treatment proportions were 45.4% and 38.2%, respectively
Castro et al. [71]	120 patients (mostly locally advanced disease)	cisplatin-based CRT	1. After the commencement of therapy, 35% of patients were identified as cachectic 2. Cachectic patients demonstrated lower mid-arm muscle circumference (MAMC) and muscle strength 3. Some patients were cachectic without evident weight loss
Gosak et al. [72]	40 patients (82.5% in stage IV)	RT alone or CRT postoperative (42.5%)	1. Before RT, 20% of patients were malnourished and 7.5% cachectic; post-treatment proportions were 10% and 35%, respectively 2. Median fat-free mass index (FFMI) significantly decreased from median score of 19.17 kg/m^2^ to 18.28 kg/m^2^ in the therapy period 3. Median albumin concentration significantly decreased from median score of 44.15 g/L to 41.73 g/L in the therapy period
Kwon et al. [73]	361 patients (76.7% in stage IV)	RT, CRT, and surgery+RT (33.8%)	1. Before treatment, 6.1% of patients were cachectic; post-treatment proportions were 45% immediately after therapy end and 18.4% 6-months after therapy 2. In cause-specific survival analysis, patients with cachexia had a higher probability of cancer-specific death, non-cancerous death, and overall death
Orell et al. [74]	58 patients (84.5% in stage IV)	RT alone (6.9%) CRT (70.7%) Surgery + RT (3.4%) Surgery + CRT (19%)	1. The prevalence of malnutrition increased during treatment from 27 to 85% in the intensive nutritional counseling group and from 44 to 75% in the on-demand nutritional counseling group 2. Lower baseline muscle strength and malnutrition were associated with worse disease-free survival (DFS) 3. Poor muscle strength and malnutrition at baseline were associated with poor OS
Citak et al. [75]	54 patients (79.6% in stage III-IV)	RT alone (62.5%) CRT (37.5%)	1. Before RT, 9.3% of patients were malnourished; post-treatment proportion was 74.1% 2. Mean MAMC decreased from 24.77 cm to 22.68 cm as a result of RT 3. Fatigue, nausea-vomiting, loss of appetite, constipation, social eating, sticky saliva, nutritional supplement, and weight loss were associated with significant malnutrition
Hofto et al. [76]	209 patients (mostly locally advanced disease)	RT alone (24%) CRT (46%) Surgery + RT (26%) Surgery + CRT (4%)	1. Before RT, 15% of patients were malnourished; post-treatment proportion was 56%. One month post-treatment malnutrition rate was 54% and after 3 months post-treatment, was 30% 2. 26% patients treated with RT had an unplanned hospital admission during treatment, of which 25% were nutrition-related
Unal et al. [77]	51 patients (82.4% in stage III-IV)	adjuvant RT (37.2%) curative RT (62.8%) concomitant CTH (62.7%)	1. Before RT, 35.3% of patients were malnourished; post-treatment proportion was 64.7% 2. The percentage of weight loss positively correlated with SGA score after RT and ECOG score after RT and RT dose
Brown et al. [78]	539 patients (mostly locally advanced disease)	RT alone (18%) CRT (29%) Surgery + RT (29%) Surgery + CRT (7%)	Before RT, 44% of patients were malnourished; post-treatment proportion was 52%
**Changes in body composition**			
Willemsen et al. [79]	137 patients (81% in stage IV)	primary or adjuvant CRT	1. The incidence of muscle wasting increased during therapy from 29% to 36% 2. The mean weight loss over the course of RT was 3.7 ± 3.5 kg in which FFM covered 1.8 ± 3.7 kg and FM 1.9 ± 3.1 kg. 3. Muscle strength significantly decreased during treatment by 3.1 ± 6.0 kg 4. Muscle wasting is an independent unfavorable prognostic factor for OS [HR = 2.1]
Willemsen et al. [80]	61 patients (73.8% in stage IV)	primary CRT (76.6%) adjuvant CRT (23.4%)	1. Significant weight loss (>2%) during first 3 weeks of therapy was noted in 37.7% of patients 2. Significant fat mass (FM) loss (−8.7 ± 9.0%) was noted in the studied patients after RT 3. Fat-free mass (FFM) change during RT was −4.0 ± 4.3% 4. Unfavorable overall survival (OS) could be observed in case of FFM loss > 1% [HR 1.85]
Nazari et al. [81]	87 patients (mostly locally advanced disease)	RT (38%) CRT (62%) Surgery + CRT (13.7%)	1. Severe weight loss (≥5% during the RT course) was observed in 47.8% of patients 2. Severe weight loss was more common at higher doses and in younger patients
Powrózek et al. [82]	70 patients (75.7% in stage IV)	RTH alone (13.4%) Surgery + RT (48.5%) Surgery + CRT (22.9%)	1. Severe weight loss (≥10%) during the RT course was observed in 32.9% of patients 2. In the study group post-RT, 52.9% of patients were identified as cachectic
Jin et al. [83]	117 patients (stage III and IV)	Definitive IMRT or TOMO (tomography radiation therapy)	1. 42.7% of patients lost at least 5% of body weight during RT 2. Nutritional intervention and non-intervention groups regarding the mean values of nutritional indicators at the same time point, that before, during, and after radiation therapy, except BMI
Langius et al. [84]	1340 patients (61% in stage III-IV)	Definitive RT (51.8%) and CRT (48.2%)	1. Before RT, 70% patients had no weight loss and critical weight loss during treatment was observed in 57% of patients 2. Patients with critical weight loss had lower 5-year overall survival rates than patients without critical weight loss during RT (survival rates: 62% vs. 70%)
**Sarcopenia**			
Chauhan et al. [85]	19 male patients (stage III-IV)	Definitive CRT	1. At baseline, 31.5% of the patients had probable sarcopenia based on their total SARC-F score, with this increasing to 89.4% at the end of CRT 2. Muscle strength decreased from mean 26.1 kg to 20.4 kg during course of therapy
Hasan et al. [86]	40 patients (mainly in stage III-IV)	Definitive IMRT/Image Guided Radiation (IGRT)	1. 57.5% of patients had sarcopenia pretreatment and 85% had sarcopenia post RT 2. Decrease in BMI strongly correlated with decreased skeletal mass index (SMI), although initial BMI did not
Chamchod et al. [87]	175 patients (stage III-IV)	Definitive RT or CRT	1. Sarcopenia was diagnosed in 65 patients (37.1%) prior to RT and it was identified in 42.7% on the post-RT scans 2. Post-RT sarcopenia, outperformed weight loss and BMI-derived cachexia metrics, because loss of lean body mass can occur independently of BMI status

**Table 3:** Tools/markers used in prediction of RT-induced malnutrition, cachexia, and body composition changes in HNC patients (anthropometric measurements and clinical scales were excluded).

Tool/Marker	Role in Prediction of RT-Induced Nutritional Deficiencies
Phase angle (PA) [52]	The risk of malnutrition/cachexia developing during CRT increased by 1.71 per mean PA decrease by one unit
PA [92]	Patients with low PA had 9.3-fold higher chance of BMI reduction below 18.5 kg/m^2^ and over 5.9-fold and 4.2-fold higher chance of lean mass (LM) and FM reduction after therapy end compared with patients with a high PA value
pre-albumin [81]	Decrease of > 15% in pre-albumin level was more likely to be malnourished (OR = 2.442) after RT commencement. Pre-albumin level predicts weight loss during RT
pre-albumin [83]	The percentage of weight loss during RT negatively correlated with pre-albumin concentration, but not with other nutrition parameters
3-hydroxybutyrate (3HB) [102]	3HB is a relatively sensitive marker that allows earlier identification of the HNC at higher risk of > 10% weight loss during RT/CRT
*TNF-α−1031T/C* [103]	Patients with CC genotype had a significantly higher chance of BMI decrease < 18.5 kg/m^2^ (underweight) following RT (OR = 23.0) and lower total protein and albumin concentration in the blood compared to carriers of CT and TT genotypes
*SELP-2028 C/T* [104]	The chance of losing ≥ 10% body weight and the development of cachexia during radical RT in patients with CC and CT genotypes was five times higher than TT genotype carriers (OR = 5.0)
*PLIN113, 041A/G* [105]	The dynamics of the adipose tissue lipolysis during RT was the highest in patients with AA genotype. They lost an average of 37.01% FM, while patients with GA and GG genotypes only lost an average of 7.73% FM. The risk of losing ≥20% or ≥30% FM during RT in AA genotype carriers was over five and over two times higher, respectively, than in men with GA and GG genotypes (OR = 5.78 and OR = 2.28).
*ITGAM-323G>A* [106]	The presence of the A allele of the *ITGAM* significantly (over 14-fold) reduced the risk of severe disturbances in nutritional status assessed according to the SGA scale (OR = 0.07) during RT
miRNA-130a [78]	Patients with low miRNA expression had over a three-fold higher risk of BMI decrease below 18.5, over six-fold higher risk of losing over 5% of body weight, and higher risk of > 10% weight reduction OR = 14.18, after the RT
miRNA-181a + PA [107]	Patients with simultaneous presence of low PA and high miRNA expression were at a significantly higher risk of decreasing the FFMI < 14.9 kg/m^2^ (OR = 5.14), FFM < 44.7 kg (OR = 6.20), and lean mass (OR = 10.0) during RT

## Data Availability

Not applicable.

## References

[1] Cohen N., Fedewa S., Chen A.Y. (2018). Epidemiology and Demographics of the Head and Neck Cancer Population. Oral Maxillofac. Surg. Clin. N. Am..

[2] Vigneswaran N., Williams M.D. (2014). Epidemiologic Trends in Head and Neck Cancer and Aids in Diagnosis. Oral Maxillofac. Surg. Clin. N. Am..

[3] World Health Organization Cancer. http://www.who.int/selection_medicines/committees/expert/20/applications/HeadNeck.pdf.

[4] Bray F., Ferlay J., Soerjomataram I., Siegel R.L., Torre L.A., Jemal A. (2018). Global cancer statistics 2018: GLOBOCAN estimates of incidence and mortality worldwide for 36 cancers in 185 countries. CA Cancer J. Clin..

[5] Siegel R.L., Miller K.D., Jemal A. (2020). Cancer statistics, 2020. CA Cancer J. Clin..

[6] Jemal A., Siegel R., Xu J., Ward E. (2010). Cancer Statistics, 2010. CA Cancer J. Clin..

[7] Pulte D., Brenner H. (2010). Changes in Survival in Head and Neck Cancers in the Late 20th and Early 21st Century: A Period Analysis. Oncologist.

[8] Laura Q.M., Chow M.D. (2020). Head and Neck cancer. N. Engl. J. Med..

[9] Dolan R.W., Vaughan C.W., Fuleihan N. (1998). Symptoms in early head and neck cancer: An inadequate indicator. Otolaryngol. Neck Surg..

[10] Golusinski W., Kubiak A., Trojanowski M., Korytowska A., Pietrysiak A., Manasterski J., Pychyński T., Golusinski P., Majchrzak E., Sówka M. (2015). National Programme for Prevention and Early Detection of Head and Neck Cancer. Otolaryngol. Polska.

[11] Dhull A.K., Atri R., Dhankhar R., Chauhan A.K., Kaushal V. (2018). Major Risk Factors in Head and Neck Cancer: A Retrospective Analysis of 12-Year Experiences. World J. Oncol..

[12] Economopoulou P., Kotsantis I., Psyrri A. (2020). Special Issue about Head and Neck Cancers: HPV Positive Cancers. Int. J. Mol. Sci..

[13] Lacko M., Braakhuis B.J.M., Sturgis E.M., Boedeker C.C., Suárez C., Rinaldo A., Ferlito A., Takes R.P. (2014). Genetic Susceptibility to Head and Neck Squamous Cell Carcinoma. Int. J. Radiat. Oncol..

[14] Koyfman S.A., Post T.W. (2018). General principles of radiation therapy for head and neck cancer. UpToDate.

[15] Semrau R. (2017). The Role of Radiotherapy in the Definitive and Postoperative Treatment of Advanced Head and Neck Cancer. Oncol. Res. Treat..

[16] Yeh S.-A. (2010). Radiotherapy for Head and Neck Cancer. Semin. Plast. Surg..

[17] Gutiontov S.I., Shin E.J., Lok B., Lee N.Y., Cabanillas R. (2016). Intensity-Modulated Radiation Therapy (IMRT) for Head and Neck Surgeons. Head Neck.

[18] Siddiqui F., Movsas B. (2017). Management of Radiation Toxicity in Head and Neck Cancers. Semin. Radiat. Oncol..

[19] Tolentino E.D.S., Centurion B.S., Ferreira L.H.C., De Souza A.P., Damante J.H., Rubira-Bullen I.R.F. (2011). Oral adverse effects of head and neck radiotherapy: Literature review and suggestion of a clinical oral care guideline for irradiated patients. J. Appl. Oral Sci..

[20] Muzumder S., Srikantia N., Udayashankar A.H., Kainthaje P.B., Sebastian M.G. (2019). Burden of acute toxicities in head-and-neck radiation therapy: A single-institutional experience. S. Asian J. Cancer.

[21] Baracos V.E., Martin L., Korc M., Guttridge D.C., Fearon K. (2018). Cancer-associated cachexia. Nat. Rev. Dis. Prim..

[22] Fox K.M., Brooks J.M., Gandra S.R., Markus R., Chiou C.F. (2009). Estimation of Cachexia among Cancer Patients Based on Four Definitions. J. Oncol..

[23] Cederholm T., Barazzoni R., Austin P., Ballmer P., Biolo G., Bischoff S.C., Compher C., Correia I., Higashiguchi T., Holst M. (2017). ESPEN guidelines on definitions and terminology of clinical nutrition. Clin. Nutr..

[24] Evans W.J., Morley J.E., Argilés J., Bales C., Baracos V., Guttridge D., Jatoi A., Kalantar-Zadeh K., Lochs H., Mantovani G. (2008). Cachexia: A new definition. Clin. Nutr..

[25] Fearon K., Strasser F., Anker S.D., Bosaeus I., Bruera E., Fainsinger R.L., Jatoi A., Loprinzi C., Macdonald N., Mantovani G. (2011). Definition and classification of cancer cachexia: An international consensus. Lancet Oncol..

[26] Arends J.J., Baracos V.V., Bertz H.H., Bozzetti F., Calder P.P., Deutz N., Erickson N.N., Laviano A.A., Lisanti M.M., Lobo D.N.D. (2017). ESPEN expert group recommendations for action against cancer-related malnutrition. Clin. Nutr..

[27] Anker S.D., Coats A.J.S., Morley J.E., Rosano G., Bernabei R., Von Haehling S., Kalantar-Zadeh K. (2014). Muscle wasting disease: A proposal for a new disease classification. J. Cachex- Sarcopenia Muscle.

[28] Aapro M., Arends J., Bozzetti F., Fearon K.C.H., Grunberg S.M., Herrstedt J., Hopkinson J., Jacquelin-Ravel N., Jatoi A., Kaasa S. (2014). Early recognition of malnutrition and cachexia in the cancer patient: A position paper of a European School of Oncology Task Force. Ann. Oncol..

[29] Muscaritoli M., Anker S., Argilés J., Aversa Z., Bauer J., Biolo G., Boirie Y., Bosaeus I., Cederholm T., Costelli P. (2010). Consensus definition of sarcopenia, cachexia and pre-cachexia: Joint document elaborated by Special Interest Groups (SIG) “cachexia-anorexia in chronic wasting diseases” and “nutrition in geriatrics”. Clin. Nutr..

[30] Yoshida T., Delafontaine P. (2015). Mechanisms of Cachexia in Chronic Disease States. Am. J. Med Sci..

[31] Donohoe C.L., Ryan A.M., Reynolds J.V. (2011). Cancer Cachexia: Mechanisms and Clinical Implications. Gastroenterol. Res. Pr..

[32] Seyfried T.N., Shelton L.M. (2010). Cancer as a metabolic disease. Nutr. Metab..

[33] Tisdale M.J. (2009). Mechanisms of Cancer Cachexia. Physiol. Rev..

[34] Petruzzelli M., Wagner E.F. (2016). Mechanisms of metabolic dysfunction in cancer-associated cachexia. Genes Dev..

[35] De Matos-Neto E.M., Lima J.D., de Pereira W.O., Figuerêdo R.G., Riccardi D.M.D.R., Radloff K., das Neves R.X., Camargo R.G., Maximiano L.F., Tokeshi F. (2015). Systemic Inflammation in Cachexia—Is Tumor Cytokine Expression Profile the Culprit?. Front. Immunol..

[36] Porporato P. (2016). Understanding cachexia as a cancer metabolism syndrome. Oncogenesis.

[37] Seelaender M.C., Laviano A., Busquets S., Püschel G.P., Margaria T., Batista M.L. (2015). Inflammation in Cachexia. Mediat. Inflamm..

[38] www.biorender.com.

[39] Naumann P., Eberlein J., Farnia B., Liermann J., Hackert T., Debus J.E., Combs S. (2019). Cachectic Body Composition and Inflammatory Markers Portend a Poor Prognosis in Patients with Locally Advanced Pancreatic Cancer Treated with Chemoradiation. Cancers.

[40] Ni J., Zhang L. (2020). Cancer Cachexia: Definition, Staging, and Emerging Treatments. Cancer Manag. Res..

[41] Grundmann O., Yoon S.L., Williams J.J., Preedy V., Patel V. (2019). Malnutrition, Cachexia, and Quality of Life in Patients with Cancer. Handbook of Famine, Starvation, and Nutrient Deprivation.

[42] Von Haehling S., Anker S.D. (2010). Cachexia as a major underestimated and unmet medical need: Facts and numbers. J. Cachexia Sarcopenia Muscle.

[43] Penet M.-F., Bhujwalla Z.M. (2015). Cancer Cachexia, Recent Advances, and Future Directions. Cancer J..

[44] Von Haehling S., Anker S.D. (2014). Prevalence, incidence and clinical impact of cachexia: Facts and numbers-update 2014. J. Cachexia Sarcopenia Muscle.

[45] Von Meyenfeldt M. (2005). Cancer-associated malnutrition: An introduction. Eur. J. Oncol. Nurs..

[46] Andreoli A., De Lorenzo A., Cadeddu F., Iacopino L., Grande M. (2011). New trends in nutritional status assessment of cancer patients. Eur. Rev. Med. Pharmacol. Sci..

[47] Dev R. (2018). Measuring cachexia-diagnostic criteria. Ann. Palliat. Med..

[48] Sadeghi M., Keshavarz-Fathi M., Baracos V., Arends J., Mahmoudi M., Rezaei N. (2018). Cancer cachexia: Diagnosis, assessment, and treatment. Crit. Rev. Oncol..

[49] Jager-Wittenaar H., Dijkstra P.U., Dijkstra G., Bijzet J., Langendijk J.A., Van Der Laan B.F.A.M., Roodenburg J.L.N. (2017). High prevalence of cachexia in newly diagnosed head and neck cancer patients: An exploratory study. Nutrition.

[50] Richey L.M., George J.R., Couch M.E., Kanapkey B.K., Yin X., Cannon T., Shores C.G. (2007). Defining Cancer Cachexia in Head and Neck Squamous Cell Carcinoma. Clin. Cancer Res..

[51] Orell-Kotikangas H., Österlund P., Mäkitie O., Saarilahti K., Ravasco P., Schwab U., Mäkitie A.A. (2017). Cachexia at diagnosis is associated with poor survival in head and neck cancer patients. Acta Oto Laryngol..

[52] Małecka-Massalska T., Smolen A., Morshed K. (2013). Extracellular–to–body cell mass ratio and subjective global assessment in head-and-neck cancers. Curr. Oncol..

[53] Stegel P., Kozjek N.R.A., Brumen B., Strojan P. (2016). Bioelectrical impedance phase angle as indicator and predictor of cachexia in head and neck cancer patients treated with (chemo)radiotherapy. Eur. J. Clin. Nutr..

[54] Kang W.-X., Li W., Huang S., Dang Y., Gao H. (2016). Effects of nutritional intervention in head and neck cancer patients undergoing radiotherapy: A prospective randomized clinical trial. Mol. Clin. Oncol..

[55] Yanni A., Dequanter D., Lechien J.R., Loeb I., Rodriguez A., Javadian R., Van Gossum M. (2019). Malnutrition in head and neck cancer patients: Impacts andindications of a prophylactic percutaneous endoscopic gastrostomy. Eur. Ann. Otorhinolaryngol. Head Neck Dis..

[56] Ling H.H., Yeh K.Y., Ng S.H., Wang C.H., Lai C.H., Wu T.H., Chang P.H., Chou W.C., Chen F.P., Lin Y.C. (2020). Determining Malnutrition Assessment Criteria to Predict One-Year Mortality for Locally Advanced Head and Neck Cancer Patients Undergoing Concurrent Chemoradiotherapy. Nutrients.

[57] Van Bokhorst–de van der Schueren M., van Leeuwen P., Sauerwein H., Kuik D., Snow G.B., Quak J.J. (1997). Assessment of malnutrition parameters in head and neck cancer and their relation to postoperative complications. Head Neck.

[58] Neoh M.K., Malaysia U.P., Abu Zaid Z., Rahman Z.A., Jamhuri N., Kahairudin Z., Samwil S.N.A., Abdullah A., Ho C.Y., Lai B.S.H. (2020). Factors associated with malnutrition among head and neck cancer in-patients before radiotherapy in National Cancer Institute, Putrajaya. Malays. J. Nutr..

[59] Silva P.B., Ramos G.H.A., Petterle R.R., Borba V.Z.C. (2021). Sarcopenia as an early complication of patients with head and neck cancer with dysphagia. Eur. J. Cancer Care.

[60] Ganju R.G., Morse R., Hoover A., TenNapel M., Lominska C. (2019). The impact of sarcopenia on tolerance of radiation and outcome in patients with head and neck cancer receiving chemoradiation. Radiother. Oncol..

[61] Langius J.A., Doornaert P., Spreeuwenberg M.D., Langendijk J.A., Leemans C.R. (2010). Radiotherapy on the neck nodes predicts severe weight loss in patients with early stage laryngeal cancer. Radiother. Oncol..

[62] Unsal D., Mentes B., Akmansu M., Uner A., Oguz M., Pak Y. (2006). Evaluation of nutritional status in cancer patients receiving radiotherapy: A prospective study. Am. J. Clin. Oncol..

[63] Gorenc M., Kozjek N.R., Strojan P. (2015). Malnutrition and cachexia in patients with head and neck cancer treated with (chemo)radiotherapy. Rep. Pract. Oncol. Radiother..

[64] Haghjoo S. (2015). Malnutrition associated with head and neck cancers. Rev. Clin. Med..

[65] Almada-Correia I., Neves P.M., Mäkitie A., Ravasco P. (2019). Body Composition Evaluation in Head and Neck Cancer Patients: A Review. Front. Oncol..

[66] Findlay M., White K., Stapleton N., Bauer J. (2020). Is sarcopenia a predictor of prognosis for patients undergoing radiotherapy for head and neck cancer? A meta-analysis. Clin. Nutr..

[67] Koom W.S., Ahn S.D., Song S.Y., Lee C.G., Moon S.H., Chie E.K., Jang H.S., Oh Y.-T., Lee H.S., Keum K.C. (2012). Nutritional status of patients treated with radiotherapy as determined by subjective global assessment. Radiat. Oncol. J..

[68] Planas M., Álvarez-Hernández J., Celaya-Pérez S., Araujo K., De Lorenzo A.G., on behalf of the PREDyCES^®^ Researchers (2016). Prevalence of hospital malnutrition in cancer patients: A sub-analysis of the PREDyCES^®^ study. Support. Care Cancer.

[69] Barker L.A., Gout B.S., Crowe T.C. (2011). Hospital Malnutrition: Prevalence, Identification and Impact on Patients and the Healthcare System. Int. J. Environ. Res. Public Health.

[70] Kang M.C., Kim J.T., Ryu S., Moon J.Y., Park J.H., Park J.K., Baik H.W., Seo J.-M., Son M.W., Song G.A. (2018). Prevalence of Malnutrition in Hospitalized Patients: A Multicenter Cross-sectional Study. J. Korean Med Sci..

[71] Castro G., Das Neves W., Rivelli T.G., Simao E.F., Martins R.E., Kulcsar M.A.V. (2019). Cachexia in head and neck squamous cell carcinoma (HNSCC) patients (pts) after cisplatin-based chemoradiation (CRT): A cross-sectional study. J. Clin. Oncol..

[72] Gosak M., Gradišar K., Kozjek N.R., Strojan P. (2020). Psychological distress and nutritional status in head and neck cancer patients: A pilot study. Eur. Arch. Oto Rhino Laryngol..

[73] Kwon M., Kim R.B., Roh J.-L., Lee S., Kim S.-B., Choi S.-H., Nam S.Y., Kim S.Y. (2016). Prevalence and clinical significance of cancer cachexia based on time from treatment in advanced-stage head and neck squamous cell carcinoma. Head Neck.

[74] Orell H., Schwab U., Saarilahti K., Osterlund P., Ravasco P., Makitie A. (2019). Nutritional Counseling for Head and Neck Cancer Patients Undergoing (Chemo) Radiotherapy—A Prospective Randomized Trial. Front. Nutr..

[75] Citak E., Tulek Z., Uzel O. (2018). Nutritional status in patients with head and neck cancer undergoing radiotherapy: A longitudinal study. Support. Care Cancer.

[76] Hofto S., Abbott J., Jackson J.E., Isenring E. (2018). Investigating adherence to Australian nutritional care guidelines in patients with head and neck cancer. Cancers Head Neck.

[77] Unal D., Orhan O., Eroglu C., Kaplan B. (2013). Prealbumin is a more sensitive marker than albumin to assess the nutritional status in patients undergoing radiotherapy for head and neck cancer. Współczesna Onkolog..

[78] Brown T., Ross L.J., Jones L., Hughes B., Banks M. (2014). Nutrition outcomes following implementation of validated swallowing and nutrition guidelines for patients with head and neck cancer. Support. Care Cancer.

[79] Willemsen A.C.H., Degens J.H.R.J., Baijens L.W.J., Dingemans A.C., Hoeben A., Hoebers F.J.P., De Ruysscher D.K.M., Schols A.M.W.J. (2020). Early Loss of Fat Mass During Chemoradiotherapy Predicts Overall Survival in Locally Advanced Squamous Cell Carcinoma of the Lung, but Not in Locally Advanced Squamous Cell Carcinoma of the Head and Neck. Front. Nutr..

[80] Willemsen A., Hoeben A., Lalisang R.I., Van Helvoort A., Wesseling F.W., Hoebers F., Baijens L.W.J., Schols A. (2020). Disease-induced and treatment-induced alterations in body composition in locally advanced head and neck squamous cell carcinoma. J. Cachex Sarcopenia Muscle.

[81] Nazari V., Pashaki A.S., Hasanzadeh E. (2021). The reliable predictors of severe weight loss during the radiotherapy of Head and Neck Cancer. Cancer Treat. Res. Commun..

[82] Powrózek T., Mlak R., Brzozowska A., Mazurek M., Gołębiowski P., Małecka-Massalska T. (2018). miRNA-130a Significantly Improves Accuracy of SGA Nutritional Assessment Tool in Prediction of Malnutrition and Cachexia in Radiotherapy-Treated Head and Neck Cancer Patients. Cancers.

[83] Pei-Jing L., Li K.-X., Li P.-J., Huang S., Chen X.-Z., Chen M., Hu Q.-Y., Shi L., Chen Y.-Y. (2017). An evaluation of nutrition intervention during radiation therapy in patients with locoregionally advanced nasopharyngeal carcinoma. Oncotarget.

[84] Langius J.A.E., Bakker S.J., Rietveld D.H.F., Kruizenga H.M.A., Langendijk J., Weijs P.J.M., Leemans C.R. (2013). Critical weight loss is a major prognostic indicator for disease-specific survival in patients with head and neck cancer receiving radiotherapy. Br. J. Cancer.

[85] Chauhan N.S., Samuel S.R., Meenar N., Saxena P.U., Keogh J. (2020). Sarcopenia in male patients with head and neck cancer receiving chemoradiotherapy: A longitudinal pilot study. PeerJ.

[86] Hasan S., Miranda D., Landau E., Ferndandez E., Leiberfarb M. (2014). Sarcopenia in Head-and-Neck Cancer: A Significant Problem in Patients Receiving Intensity Modulated (IMRT) and Image Guided Radiation (IGRT) as Assessed by a Validated CT-Based Assessment Tool Epidemiology and Prevention. Int. J. Radiat. Oncol. Biol. Phys..

[87] Chamchod S., Fuller C.D., Grossberg A.J., Mohamed A.S., Heukelom J., Eichelberger H. (2015). Sarcopenia/cachexia is associated with reduced survival and locoregional control in head and neck cancer patients receiving radiotherapy: Results from quantitative imaging analysis of lean body mass. Oncology.

[88] Santarpia L., Contaldo F., Pasanisi F. (2011). Nutritional screening and early treatment of malnutrition in cancer patients. J. Cache Sarcopenia Muscle.

[89] Ferguson M., Bauer J., Gallagher B., Capra S., Christie D., Mason B.R. (1999). Validation of a malnutrition screening tool for patients receiving radiotherapy. Australas. Radiol..

[90] Leuenberger M., Kurmann S., Stanga Z. (2010). Nutritional screening tools in daily clinical practice: The focus on cancer. Support. Care Cancer.

[91] Tang P.L., Wang H.H., Lin H.S., Liu W.S., Chen M.L., Chou F.H. (2018). Body Composition Early Identifies Cancer Patients with Radiotherapy at Risk for Malnutrition. J. Pain Symptom. Manag..

[92] Małecka-Massalska T., Powrózek T., Prendecka M., Mlak R., Sobieszek G., Brzozowski W., Brzozowska A. (2019). Phase Angle as an Objective and Predictive Factor of Radiotherapy-induced Changes in Body Composition of Male Patients With Head and Neck Cancer. Vivo.

[93] Meerkerk C.D., Chargi N., De Jong P.A., Bos F.V.D., De Bree R. (2020). Sarcopenia measured with handgrip strength and skeletal muscle mass to assess frailty in older patients with head and neck cancer. J. Geriatr. Oncol..

[94] Ufuk F., Herek D., Yüksel D. (2019). Diagnosis of Sarcopenia in Head and Neck Computed Tomography: Cervical Muscle Mass as a Strong Indicator of Sarcopenia. Clin. Exp. Otorhinolaryngol..

[95] Nishikawa D., Hanai N., Suzuki H., Koide Y., Beppu S., Hasegawa Y. (2018). The Impact of Skeletal Muscle Depletion on Head and Neck Squamous Cell Carcinoma. ORL.

[96] Sokolenko A., Imyanitov E.N. (2018). Molecular Diagnostics in Clinical Oncology. Front. Mol. Biosci..

[97] Loumaye A., Thissen J.-P. (2017). Biomarkers of cancer cachexia. Clin. Biochem..

[98] Johns N., Tan B.H., MacMillan M. (2014). Genetic basis of interindividual susceptibility to cancer cachexia: Selection of potential candidate gene polymorphisms for association studies. J. Genet..

[99] Freire P.P., Fernandez G.J., Cury S.S., De Moraes D., Oliveira J.S., De Oliveira G., Dal-Pai-Silva M., Dos Reis P.P., Carvalho R.F. (2019). The Pathway to Cancer Cachexia: MicroRNA-Regulated Networks in Muscle Wasting Based on Integrative Meta-Analysis. Int. J. Mol. Sci..

[100] Donzelli S., Farneti A., Marucci L., Ganci F., Sacconi A., Strano S., Sanguineti G., Blandino G. (2020). Non-coding RNAs as Putative Biomarkers of Cancer-Associated Cachexia. Front. Cell. Dev. Biol..

[101] Summerer I., Niyazi M., Unger K., Pitea A., Zangen V., Hess J., Atkinson M.J., Belka C., Moertl S., Zitzelsberger H. (2013). Changes in circulating microRNAs after radiochemotherapy in head and neck cancer patients. Radiat. Oncol..

[102] Bieleń A., Mrochem-Kwarciak J., Skorupa A., Ciszek M., Heyda A., Wygoda A., Kotylak A., Składowski K., Sokół M. (2019). NMR-based metabolomics in real-time monitoring of treatment induced toxicity and cachexia in head and neck cancer: A method for early detection of high risk patients. Metabolomics.

[103] Powrózek T., Mlak R., Brzozowska A., Mazurek M., Gołębiowski P., Małecka-Massalska T. (2018). Relationship between TNF-α −1031T/C gene polymorphism, plasma level of TNF-α, and risk of cachexia in head and neck cancer patients. J. Cancer Res. Clin. Oncol..

[104] Powrózek T., Mlak R., Brzozowska A., Mazurek M., Gołębiowski P., Małecka-Massalska T. (2019). Relationship Between -2028 C/T SELP Gene Polymorphism, Concentration of Plasma P-Selectin and Risk of Malnutrition in Head and Neck Cancer Patients. Pathol. Oncol. Res..

[105] Powrózek T., Brzozowska A., Mazurek M., Prendecka M., Homa-Mlak I., Mlak R., Małecka-Massalska T. (2020). AA genotype of PLIN1 13041A>G as an unfavourable predictive factor of malnutrition associated with fat mass loss in locally advanced head and neck cancer male patients treated with radiotherapy. Support. Care Cancer.

[106] Mazurek M., Mlak R., Homa-Mlak I., Powrózek T., Brzozowska A., Gołębiowski P., Małecka-Massalska T. (2020). Polymorphism of The Regulatory Region of the *ITGAM* Gene (-323G>A) as a Novel Predictor of a Poor Nutritional Status in Head and Neck Cancer Patients Subjected to Intensity-Modulated Radiation Therapy. J. Clin. Med..

[107] Powrózek T., Brzozowska A., Mazurek M., Mlak R., Sobieszek G., Małecka-Massalska T. (2019). Combined analysis of miRNA-181a with phase angle derived from bioelectrical impedance predicts radiotherapy-induced changes in body composition and survival of male patients with head and neck cancer. Head Neck.

[108] Da Rocha R.G., Santos E.M.S., Santos E.M., Gomes E.S.B., Ramos G.V., Aguiar K.M., Gonçalves B.R., Santos S.H.S., De Paula A.M.B., Guimarães A.L.S. (2019). Leptin impairs the therapeutic effect of ionizing radiation in oral squamous cell carcinoma cells. J. Oral Pathol. Med..

[109] Van Rijn-Dekker M.I., van den Bosch L., van den Hoek J.G., Bijl H.P., van Aken E.S., van der Hoorn A., Oosting S.F., Halmos G.B., Witjes M.J., van der Laan H.P. (2020). Impact of sarcopenia on survival and late toxicity in head and neck cancer patients treated with radiotherapy. Radiother. Oncol..

[110] Vagnildhaug O.M., Brunelli C., Hjermstad M.J., Strasser F., Baracos V., Wilcock A., Nabal-Vicuña M., Kaasa S., Laird B.J., Solheim O. (2019). A prospective study examining cachexia predictors in patients with incurable cancer. BMC Palliat. Care.

[111] Castillo-Martínez L., Castro-Eguiluz D., Copca-Mendoza E.T., Pérez-Camargo D.A., Reyes-Torres C.A., Damasco-Ávila E.A., López-Córdova G., Fuentes-Hernández M.R., Cetina-Pérez L., Milke-García M.D.P. (2018). Nutritional Assessment Tools for the Identification of Malnutrition and Nutritional Risk Associated with Cancer Treatment. Rev. Invest. Clin..

[112] González-Rodríguez M., Villar-Taibo R., Fernández-Pombo A., Pazos-Couselo M., Sifontes-Dubón M.A., Ferreiro-Fariña S., Cantón-Blanco A., Martínez-Olmos M.A. (2020). Early versus conventional nutritional intervention in head and neck cancer patients before radiotherapy: Benefits of a fast-track circuit. Eur. J. Clin. Nutr..

[113] Arribas L., Hurtós L., Taberna M., Peiró I., Vilajosana E., Lozano A., Vazquez S., Mesia R., Virgili N. (2017). Nutritional changes in patients with locally advanced head and neck cancer during treatment. Oral Oncol..

[114] Nesemeier R., Dunlap N., McClave S.A., Tennant P. (2017). Evidence-Based Support for Nutrition Therapy in Head and Neck Cancer. Curr. Surg. Rep..

[115] Arends J., Bachmann P., Baracos V., Barthelemy N., Bertz H., Bozzetti F., Fearon K., Hütterer E., Isenring E., Kaasa S. (2017). ESPEN guidelines on nutrition in cancer patients. Clin. Nutr..

[116] Trotti A.A., Bellm L., Epstein J.B., Frame D., Fuchs H.J., Gwede C.K., Komaroff E., Nalysnyk L., Zilberberg M.D. (2003). Mucositis incidence, severity and associated outcomes in patients with head and neck cancer receiving radiotherapy with or without chemotherapy: A systematic literature review. Radiother. Oncol..

[117] Paccagnella A., Morello M., Da Mosto M.C., Baruffi C., Marcon M.L., Gava A., Baggio V., Lamon S., Babare R., Rosti G. (2009). Early nutritional intervention improves treatment tolerance and outcomes in head and neck cancer patients undergoing concurrent chemoradiotherapy. Support. Care Cancer.

[118] Baxi S.S., Schwitzer E., Jones L.W. (2016). A review of weight loss and sarcopenia in patients with head and neck cancer treated with chemoradiation. Cancers Head Neck.

[119] Bye A., Sandmæl J.A., Stene G.B., Thorsen L., Balstad T.R., Solheim T.S., Pripp A.H., Oldervoll L.M. (2020). Exercise and Nutrition Interventions in Patients with Head and Neck Cancer during Curative Treatment: A Systematic Review and Meta-Analysis. Nutrition.

